# Marine-Derived Compounds as Potential Inhibitors of Hsp90 for Anticancer and Antimicrobial Drug Development: A Comprehensive In Silico Study

**DOI:** 10.3390/molecules28248074

**Published:** 2023-12-13

**Authors:** Mebarka Ouassaf, Lotfi Bourougaa, Samiah Hamad Al-Mijalli, Emad M. Abdallah, Ajmal R. Bhat, Sarkar M. A. Kawsar

**Affiliations:** 1 Group of Computational and Medicinal Chemistry, LMCE Laboratory, University of Biskra, Biskra 707000, Algeria; lotfi.bourougaa@univ-biskra.dz; 2 Department of Biology, College of Sciences, Princess Nourah Bint Abdulrahman University, Riyadh 11671, Saudi Arabia; 3 Department of Science Laboratories, College of Science and Arts, Qassim University, Ar Rass 51921, Saudi Arabia; 140208@qu.edu.sa; 4 Department of Chemistry, RTM Nagpur University, Nagpur 440033, India; bhatajmal@gmail.com; 5 Laboratory of Carbohydrate and Nucleoside Chemistry, Department of Chemistry, Faculty of Science, University of Chittagong, Chittagong 4331, Bangladesh; akawsarabe@yahoo.com

**Keywords:** anticancer, anti-bacterial agents, marine bioactive molecules, drug discovery, docking simulations, ADME, molecular dynamics simulations

## Abstract

Marine compounds constitute a diverse and invaluable resource for the discovery of bioactive substances with promising applications in the pharmaceutical development of anti-inflammatory and antibacterial agents. In this study, a comprehensive methodology was employed, encompassing pharmacophore modeling, virtual screening, in silico ADMET assessment (encompassing aspects of absorption, distribution, metabolism, excretion, and toxicity), and molecular dynamics simulations. These methods were applied to identify new inhibitors targeting the Hsp90 protein (heat shock protein 90), commencing with a diverse assembly of compounds sourced from marine origins. During the virtual screening phase, an extensive exploration was conducted on a dataset comprising 31,488 compounds sourced from the CMNPD database, characterized by a wide array of molecular structures. The principal objective was the development of structure-based pharmacophore models, a valuable approach when the pool of known ligands is limited. The pharmacophore model DDRRR was successfully constructed within the active sites of the Hsp90 crystal structure. Subsequent docking studies led to the identification of six compounds (CMNPD **22591**, **9335**, **10015**, **360799**, **15115**, and **20988**) demonstrating substantial binding affinities, each with values below −8.3 kcal/mol. In the realm of in silico ADMET predictions, five of these compounds exhibited favorable pharmacokinetic properties. Furthermore, molecular dynamics simulations and total binding energy calculations using MM-PBSA indicated that these marine-derived compounds formed exceptionally stable complexes with the Hsp90 receptor over a 100-nanosecond simulation period. These findings underscore the considerable potential of these novel marine compounds as promising candidates for anticancer and antimicrobial drug development.

## 1. Introduction

In the present era, modern medicine grapples with significant challenges. The looming prospect of a precipitous decline in the efficacy of contemporary medical treatments is rapidly materializing. It has been widely acknowledged that there is an imperative need for the innovation of new antibiotics aimed at averting this impending crisis. Nevertheless, the persistent emergence of highly resistant microbial strains, often referred to as “superbugs,” persists unabated, thereby exacerbating the likelihood of a widespread epidemic characterized by profound antibiotic resistance, which constitutes a formidable and imminent menace to global public health [1,2]. On the other side, the process of designing anticancer drugs is widely recognized as a complex, costly, time-intensive, and formidable endeavor. Despite advancements in tumor biotechnology, the innovation of efficacious anticancer medications remains a strenuous and time-consuming undertaking and necessitates robust multidisciplinary partnerships, encompassing expertise in medicinal chemistry, computational chemistry, biology, pharmacology, and clinical research [3,4].

In recent years, marine bioactive compounds have attracted scientific attention due to their therapeutic potential for numerous medical conditions. Marine natural products manifest a diverse array of pharmaceutically relevant bioactivities, encompassing antibiotic, antiviral, neurodegenerative, anticancer, and anti-inflammatory properties [5,6,7]. Numerous investigators have extracted anticancer compounds from a range of marine organisms, including cyanobacteria, fungi, sponges, tunicates, ascidians, mollusks, and fish. Anticancer peptides elicit cell death via a distinctive mechanism characterized by their selectivity toward cancer cells and their role in inducing membrane disintegration [8]. Marine natural products represent a valuable reservoir of chemically varied compounds. Several of these marine products, characterized by their biological diversity and therapeutic potential, have demonstrated substantial antimicrobial activity against a variety of pathogenic microorganisms [9].

Heat shock protein 90 (Hsp90) is a family of ATP-dependent molecular chaperones that play a critical role in regulating the stability and function of client proteins involved in cellular stress [10]. Heat shock protein 90 (Hsp90) serves as a molecular chaperone essential for maintaining the stability and functionality of several signaling proteins that are conditionally activated or expressed. The rational application of Hsp90 inhibitors, either as monotherapies or in conjunction with other pharmaceutical agents, holds the potential to enhance the therapeutic strategies for addressing various types of cancer [11]. Interestingly, Hsp90 inhibitors could be used for antibiotic targeting. Bacteria employ two-component signaling systems to adapt to alterations in their surroundings, and these systems are marked by their high degree of conservation and reliance on histidine kinases. Targeting histidine kinases for inhibition holds the potential for exerting a broad-spectrum antimicrobial effect. Notably, the ATP binding domain of histidine kinases shares conservation with the ATPase domain found in eukaryotic Hsp90 molecular chaperones [12].

In eukaryotic cells, Hsp90 is involved in various cellular processes, including protein folding, maturation, and degradation. Hsp90 family proteins consist of four paralogs, each residing in different subcellular locations: Hsp90-α/β in the cytoplasm, glucose-regulated protein 94 (Grp94) in the endoplasmic reticulum (ER), and tumor necrosis factor receptor-associated protein-1 (TRAP1) in mitochondria [13]. Initially, considering Hsp90 as a potential therapeutic target was challenging due to its essential role in the cytoplasm for normal cell viability and growth [14]. However, the discovery of geldanamycin (GA) and its powerful anticancer effects through Hsp90 inhibition [15,16] generated considerable interest and research in this area. As a result, a wide range of Hsp90 inhibitors have been identified and synthesized; it has been documented that benzoquinone ansamycins, a category of naturally derived antibiotics, exhibit inhibitory effects on the functioning of Hsp90 [17]. Subsequent clinical trials have assessed the impact of Hsp90 inhibitors as adjunctive therapy for various tumor types, and presently, ongoing investigations are underway to explore innovative and potentially more efficacious strategies for cancer management [18]. Hsp90 plays a pivotal role in stabilizing and activating a wide array of client proteins, exceeding 300 in number (refer to www.picard.ch/downloads/HSP90interactors.pdf accessed 6 novembre 2022). Notably, substantial portions of these Hsp90 client proteins are integral to oncogenic signaling, orchestrating key aspects of malignancy, including proliferation, evasion of apoptosis, immortalization, invasion, angiogenesis, and metastasis [19]. Inhibiting Hsp90 results in the swift inhibition of client protein activity, followed by their ubiquitin-mediated proteasomal degradation. This leads to the simultaneous depletion of multiple oncoproteins, causing a comprehensive downregulation of signals across various oncogenic pathways and influencing every facet of the malignant phenotype. Cancer cells exhibit heightened sensitivity to Hsp90 inhibition due to their dependence on the oncogenic processes that propel malignancy. Consequently, they rely on Hsp90 for the chaperoning and maintenance of these crucial oncogenic pathways [20]. Furthermore, cancer cells depend on Hsp90 to stabilize mutated, fused, and overexpressed oncoproteins such as vSRC, HER2, BCR-ABL, B-RAF, and ELM4-ALK. Hsp90 itself is often overexpressed in cancer cells and is known to exist in a highly active, multi-chaperone complex [21]. Recent attention has also been directed toward the role of secreted Hsp90 in fostering cancer cell invasion and metastasis [22]. Invasive cancer cells have been observed to release Hsp90-α, which subsequently activates the pro-invasive protein matrix metalloproteinases, contributing to heightened cancer cell migration [23]. This emerging aspect underscores the diverse functions of Hsp90 in the complex landscape of cancer biology. The search for novel Hsp90 inhibitor drugs is necessary to address the limitations and challenges associated with current therapies. Developing drugs that overcome resistance, exhibit improved safety profiles, and possess favorable pharmacokinetic properties is crucial for maximizing the therapeutic potential of Hsp90 inhibition in cancer treatment. By tackling these obstacles, we can pave the way for more effective and targeted therapies against cancer.

Drug design methodologies offer a rapid and efficient means of searching for new antibacterial or anticancer drugs. By leveraging computational tools and experimental validation, this approach enables the exploration of diverse compound libraries and facilitates the discovery of promising new therapeutic agents [24]. Natural compounds derived from marine sources, in particular, have garnered significant interest in drug discovery research. The unique marine environment offers a rich source of diverse and structurally complex compounds that possess bioactive properties, such as antibacterial, antifungal, antimalarial, anti-trypanosomal, anti-viral, anti-obesity, antitumor, and anticancer activity [25,26]. The aim of this study is to explore the potential of natural compounds derived from marine sources for drug discovery. Our focus is on tapping into the previously untapped reservoir of these compounds to uncover their pharmacological activities and therapeutic potential. We conducted an extensive investigation and screening process to identify marine-derived compounds that exhibit promising interactions with the Hsp90 protein. Utilizing computational techniques such as molecular docking, virtual screening, and pharmacophore modeling, we assessed the binding affinity and potential interactions of a diverse set of marine-derived compounds with Hsp90. Additionally, we conducted a molecular dynamics simulation using Gromacs to evaluate the stability of the formed complexes over a 100 ns timeframe, and we applied MM-PBSA calculations to validate the results of the docking study. The outcomes of our research have shown exceptionally promising results, marking a significant advancement in the quest for optimal drug candidates in the field of oncology or antimicrobial drugs.

## 2. Results

### 2.1. Generation and Validation of Target-Based Pharmacophore Model

**Figure 1:**
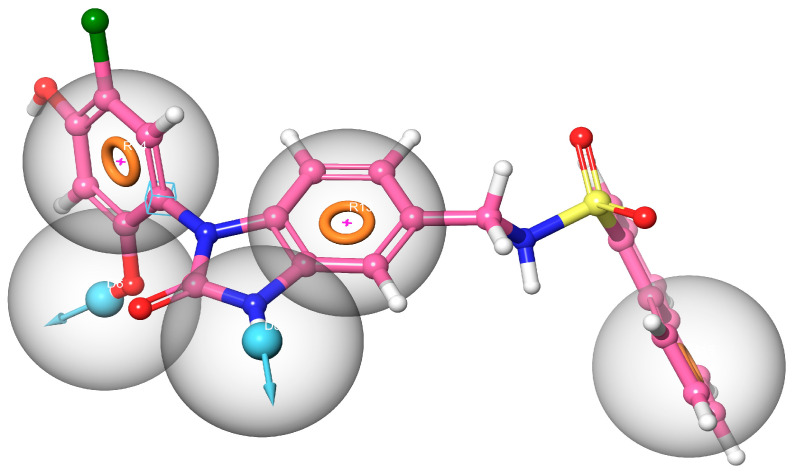
The pharmacophore model generated included HBD (blue spheres) and the aromatic center (orange sphere).

Our research is focused on identifying inhibitors of the human Hsp90 protein with the goal of addressing cancer. To achieve this, we utilized the known structure of the human Hsp90 protein and employed the Schrödinger software suite to generate a target-based pharmacophore model. The pharmacophore model was constructed based on the MEY-Hsp90 complex, which was obtained through the XP docking method. By utilizing the XP docking method, we determined the optimal binding conformation of the MEY ligand within the binding site of the Hsp90 protein. Through an extensive analysis of the interactions between the ligand and the protein, our pharmacophore model identified key features that are essential for effective inhibition, as shown in Figure 1.

This pharmacophore model DDRRR consists of five features, including two hydrogen bond donors and three hydrophobic groups. Subsequently, we applied this generated pharmacophore model DDRRR to screen marine-sourced compounds.

The validation of the predicted pharmacophore model is a crucial step in assessing its reliability in distinguishing active compounds from decoys. Established quality indi-cators such as ROC, AUC-ROC, and EF have been widely acknowledged for pharmacophore model validation [27]. The task phase in the Schrödinger software provides a screening mode for model validation by loading a set of actives and decoys into the screening window. In this study, 35 active compounds were used for model validation, and the Schrödinger database, comprising 1000 decoy structures, was employed for screening against the known actives.

The created pharmacophore template DDRRR successfully identified 29 out of 35 actives. Detailed information on total actives and decoys, as well as retrieved actives and decoys during model validation, can be found in Table S2.

RIE was computed for the hypothesis model to evaluate the ranking of active set contribution in the enrichment study. The obtained RIE value of 7.18 for the DDRRR model directed indicated its superior ranking over random distribution. To estimate the DDRRR performance, the area under the accumulation curve (AUC) of the receiver operating characteristic (ROC) curve was plotted as a reliable metric. The generated DDRRR model achieved good values of ROC (0.78) and AUC (0.86).

The receiver operating characteristic (ROC) curve for model validation illustrates specificity vs. sensitivity. The model’s high accuracy is indicated by specificity plotted on the *X* axis, representing false positives or retrieved decoys, and sensitivity on the *Y* axis, representing true positives or retrieved actives. The ROC curve in Fig S1, which deviates significantly from the dotted line representing random prediction, demonstrates the model’s predictive ability well above chance. The area under the ROC curve (AUC-ROC) and early enrichment factor (EF) values on the curve further support the model’s high predictive accuracy, sensitivity, and specificity (AUC1% = 0.84, EF1% = 19.45). Consequently, the pharmacophore model is deemed reliable for the virtual screening of CMNPD. During the screening process, we successfully identified 5156 compounds that aligned well with the model and met the criteria for the model’s 4–5 feature super-positions. The model map of the top leads is presented in Figure S2.

### 2.2. Target-Based Virtual Screening (TBVS)

In our research approach, we employed target-based virtual screening (TBVS), a widely used method in virtual screening that incorporates molecular docking techniques. Compounds that successfully passed the Lipinski drug-likeness screening, which relies on physicochemical properties to predict the drug-likeness of an oral therapeutic agent, were considered. Lipinski’s rule-of-five establishes criteria for drug-likeness, including no more than five hydrogen bond donors, no more than 10 hydrogen bond acceptors, a molecular weight not exceeding 500 Da, and an octanol–water partition coefficient (log P) not exceeding 5 [27]. Following the Lipinski analysis, compounds that demonstrated good alignment with the pharmacophore model were selected for further investigation. Our selection process involved assessing both fitness values and the number of matches, ensuring the prioritization of the most promising compounds based on the pharmacophore model. This comprehensive approach aimed to identify compounds with favorable drug-like properties and a strong alignment with the desired pharmacophoric features. The next step in the research involved conducting molecular docking studies to assess the binding interactions and affinity between the selected compounds and the target protein Hsp90. Through molecular docking, valuable insights were gained regarding the binding modes, energy scores, and potential interactions between the compounds and the Hsp90 protein.

**Figure 2:**
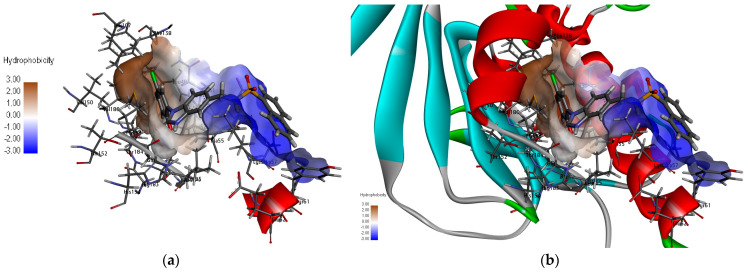
(**a**): Visualization of Hsp90 protein and the co-crystallized compound MEY, (**b**): surface representation of hydrophobic residues in the cavity.

Docking protocol validation was conducted by re-docking the co-crystallized ligand MEY (PDB: 3wod) into the catalytic domain of the Hsp90 receptor (Figure 2). The observed conformational orientation similarity between the docked pose and the co-crystallized ligand, with a root-mean-square deviation (RMSD) of 1.9857 Å, serves as confirmation of the accuracy of the docking protocol. The obtained results, as shown in Figure 2, confirm our successful identification of the correct active site of the enzyme. The binding pocket of Hsp90 was divided into two moieties: a hydrophobic moiety consisting of residues Ala55, Ile96, Met98, Leu107, Phe138, and Val150 and a hydrophilic moiety composed of residues Asn51, Asp93, and Thr184. These findings are consistent with the literature [28].

**Figure 3:**
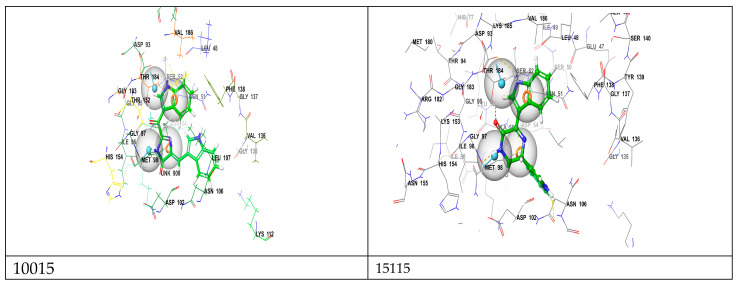

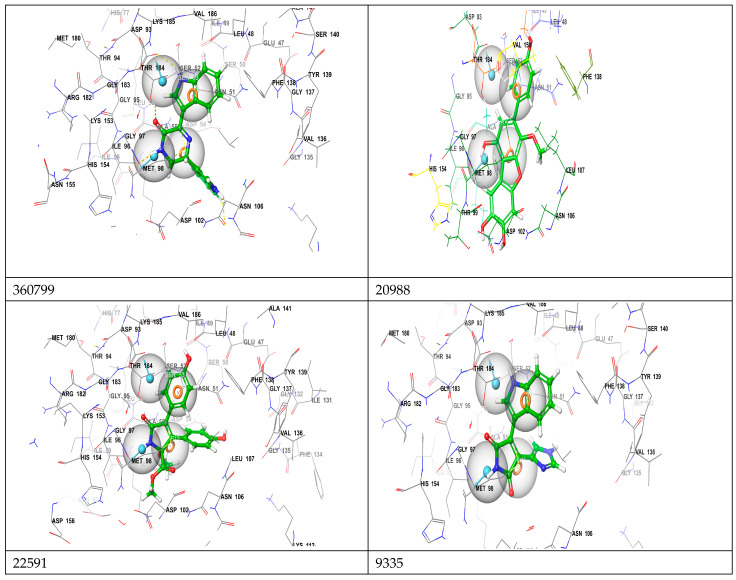
Illustrated 3d interactions between top leads within the binding site of the Hsp90 protein (PDB ID: 3OWD).

**Figure 4:**
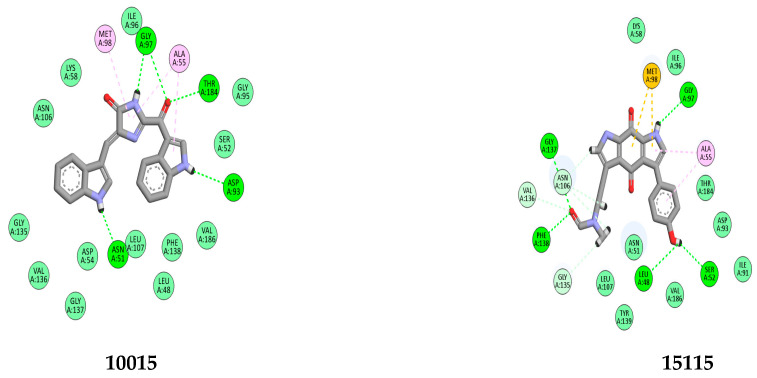

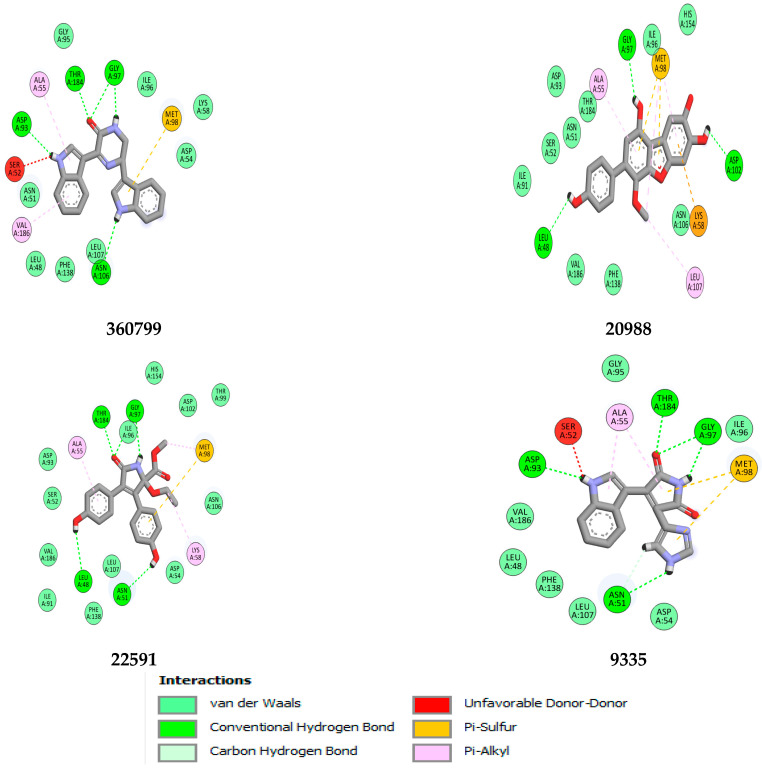
Illustrated 2d map of interactions between top leads within the binding site of the Hsp90 protein (PDB ID: 3OWD).

To enhance the accuracy and reliability of our results, we implemented a systematic approach employing two distinct docking methods: standard precision (SP) and extra precision (XP). Docking methods, such as standard precision (SP) and extra precision (XP), vary in their computational approaches and precision levels. SP docking is known for its faster computational speed and is often used for initial screenings due to its ability to process a large number of compounds efficiently. However, it sacrifices some accuracy by utilizing simplified representations and fewer computational resources. On the other hand, XP docking is characterized by its higher accuracy and more exhaustive calculations. It employs finer grid settings and conducts more thorough evaluations, making it more suitable for detailed analyses that require precise estimations of ligand–protein interactions. The choice between SP and XP docking methods is often based on research goals, with SP favored for quicker screenings and XP utilized for in-depth, accurate evaluations despite the higher computational demand [29,30]. The obtained docking scores serve as indicators of the binding affinities between each compound and the target protein. Lower scores signify stronger binding interactions between the compound and the protein. These scores can be further analyzed and interpreted to gain insights into the docking results. The docking results of our study demonstrated that six compounds, **22591**, **9335**, **10015**, **360799**, **15115**, and **20988**, achieved favorable docking scores lower than the reference ligand (SMEY = −8.32 kcal) (see Table 1). This observation suggests that these compounds exhibit greater stability and stronger binding affinity to the N-terminal ATP binding pocket of Hsp90. Our comprehensive analysis of the docking results involved visualizing the interactions within the complexes, as depicted in Figure 3 and Figure 4. To provide a detailed overview, we have compiled a summary of the interactions and their respective types in Table 2. Interestingly, the analysis of the complexes revealed a significant abundance of hydrogen bonding interactions (>5) in comparison with the reference compound. This higher number of hydrogen bonding interactions contributes to the increased stability of our compounds. Hydrogen bonds, known for their strong electrostatic nature, play a critical role in ligand—receptor binding by providing stability and reinforcing the overall strength of complex formation [31]. Specific amino acids, such as Gly97, Thr184, Asn51, and Asp93, were found to be involved in hydrogen bonding interactions with our compounds within the active site of the enzyme. Remarkably, these same amino acids were also present in the reference compound complex. This similarity further supports the notion of a conserved binding mode and shared interaction pattern between our compounds and the reference compound, thus reinforcing the reliability and validity of our docking results. In addition to hydrogen bonding, our study also identified significant hydrophobic interactions between our compounds and the enzyme. Specifically, these hydrophobic interactions involved amino acids Met98, Ala55, Asp54, and Phe138. These results are similar to those found in other studies [32]. Among the amino acids involved in the binding between our compounds and the enzyme, Met98 has been found to interact with our compounds through a specific interaction type known as pi–sulfur interaction. The pi–sulfur interaction is a noncovalent interaction. In our complexes, the ligands demonstrated this interaction, where the ligands exhibit this interaction by aligning the pi–electron system of their aromatic rings with the sulfur atom in the methionine residue. This interaction is considered favorable and contributes to the overall binding between the ligand and the protein. The pharmacophore hypothesis generated for DDRRR comprises three aromatic rings and two hydrogen bond donors (Figure 1). This hypothesis elucidates crucial protein–ligand interactions. We underscore the significance of the pyrrole and pyrazole rings in enhancing binding affinity, particularly emphasizing the pi–pi interaction with Asp54 and pi-sulfur stacking interactions with Met98. The hydrogen bond donor element signifies the importance of hydrogen bond donor interactions with Gly57 and Asp93. The presence of diverse interactions between our compounds and the protein enhances stability. Our utilization of the pharmacophore model facilitated the identification of shared features among the compounds, resulting in similar bonds at the enzyme’s active site despite their diverse structures. Following the docking study, we identified six marine-derived compounds with distinct scaffolds that are anticipated to serve as potent inhibitors of the Hsp90 protein. Detailed properties and definitions for these compounds are compiled in Table S1. While these structural differences are likely to be evident, they are expected to manifest more prominently in their unique physical and chemical properties. In the following section, we delve deeper into the study and analysis of the pharmacokinetic properties and drug-likeness of these compounds.

### 2.3. Pharmacokinetic Properties and Drug Likeness

In the next phase of our study, we focused on a comprehensive exploration of the molecular and medicinal properties of the six selected compounds (Table S1). After filtering the compounds based on Lipinski’s rule and the pharmacophore model and conducting docking studies, we analyzed their pharmacokinetic properties using ADME-Tox evaluations.

#### 2.3.1. Molecular Properties

The molecular properties of all the best leads **22591**, **9335**, **10015**, **360799**, **15115**, **20988**, and MEY, as predicted by ADMETLAB Explorer, are presented in Table 3. The selected lead compounds exhibit low molecular weights (<370) and volumes, indicating their relatively small size compared with the reference compound MEY (MW = 490). The small size of our compounds can have significant implications for their physicochemical and biological properties. The lead compounds in this study exhibit a range of numbers of rings (three to five), contributing to structural complexity and potentially enhancing their interactions with biologically significant target molecules. Moreover, the compounds display varying maximum ring sizes (9 to 14), influencing their conformation and potentially affecting binding affinity and selectivity toward specific targets. The inclusion of larger rings highlights the role of the ring structure in modulating the properties and potential therapeutic applications of these compounds. Additionally, the compounds contain different numbers of heteroatoms (five to seven), which are atoms other than carbon and hydrogen. The presence of heteroatoms enhances the compounds’ interactions with the Hsp90 protein and offers the possibility of targeting specific regions or functional groups within the protein structure. The selected compounds exhibit different levels of rigidity and flexibility. Compounds **22591**, **9335**, and **15115** show high flexibility, enabling them to effectively adapt and conform to the binding site of the Hsp90 protein. We believe this inherent flexibility plays an important role in forming stable complexes and obtaining favorable docking scores. The values that represent the logarithm of the compound’s solubility (logS), partition coefficient (logP), and distribution coefficient (logD) are shown in Table 3.

The logP value represents the compound’s partition coefficient, which measures its lipophilicity or hydrophobicity. The logP values provided in Table 3 for the selected compounds range from 1.182 to 3.869, all of which are below 5. These values indicate a moderate degree of lipophilicity for the compounds. Compounds with logP values below 5 generally have good potential for membrane permeability and absorption. This property is crucial for drugs to effectively cross biological barriers and reach their target sites. The logD values provided for the compounds indicate their distribution coefficients, which describe the balance between their hydrophilic and lipophilic properties. In the provided range (1.413–3.492), the compounds exhibit moderate to high lipophilicity. A positive logD value suggests that the compounds have a higher affinity for the lipid phase compared with the aqueous phase. This finding aligns with the logP and logS values, which also indicate the compounds’ tendency toward lipophilicity and lower solubility in water.

#### 2.3.2. Medicinal Chemistry

Table 4 presents the calculated medicinal chemistry properties for the studied compounds **22591**, **9335**, **10015**, **360799**, **15115**, and **20988**. These properties include the quantitative estimate of drug-likeness (QED), synthetic accessibility score (SA score), and several other rules of interest. The QED (quantitative estimate of drug-likeness) values provided in the table range from 0.389 to 0.620. These values represent the estimated drug-likeness of the compounds, with higher values indicating a higher likelihood of being drug-like.

The SA score is a metric used to assess the synthetic accessibility of a compound. It takes into account various factors, such as the complexity of the molecular structure and the availability of starting materials and synthetic pathways [33]. A lower SA score indicates that the compound is structurally simpler and can be synthesized more easily. In Table 4 of the study, the SA score values for the compounds range from 2.635 to 3.253. These values indicate that the compounds possess relatively good synthetic accessibility. Table 4 includes the evaluation of the lead compounds based on various rules and alerts commonly used in drug discovery. The majority of the compounds demonstrated promising drug-likeness, as they satisfied the GSK rule and the golden triangle rule [34], which assesses factors such as molecular weight, lipophilicity, hydrogen bond donors and acceptors, and their balance. However, one specific compound (**360799**) did not meet the criteria of the Pfizer rule and was consequently rejected. This rule evaluates drug-likeness based on parameters such as molecular weight, logP, hydrogen bond donors and acceptors, and the number of rotatable bonds [35]. Furthermore, none of the compounds in the study exhibited any alerts according to the PAINS rule, which identifies structural features associated with assay interference. The absence of PAINS alerts suggests that the compounds are less likely to produce misleading or false-positive results and increases their potential as meaningful hits [36]. Additionally, none of the compounds triggered alerts according to the BMS rule, which identifies potential toxicity concerns. Similarly, none of the compounds raised alerts according to the chelator rule, indicating the absence of chelation-related issues. Overall, the majority of the compounds in the table meet the criteria set by these rules, indicating their potential as drug candidates. However, it is important to consider these rules as guidelines rather than definitive determinants of a compound’s suitability for drug development, as additional factors and assessments are necessary.

#### 2.3.3. Absorption

The values listed in Table 5 represent the absorption of a substance. Caco-2 permeability is a measure of how easily a substance can pass through the Caco-2 cell monolayer, as shown in the provided Table 5 for Caco-2 permeability, and the values range from −5.172 to −4.621. The website admelab states that the optimal value is higher than −5.15 based on the given data and the specified criteria. Compounds **360799**, **20988**, and **22591** show relatively better permeability through the Caco-2 cell monolayer, while compounds **10015**, **15115**, and **9335** display relatively lower permeability.

The MDCK cell monolayer is widely used as an in vitro model to assess the permeability of substances [37]. Compounds **10015**, **360799**, **15115**, and **9335** demonstrate low permeability through the MDCK cell monolayer, as their permeability values fall within the range of <2 × 10^−6^ cm/s. This suggests that these substances have limited ability to pass through the cell monolayer, indicating potential challenges in their absorption into the body. P-glycoprotein is a membrane transporter that plays a role in the absorption and distribution of substances within the body [38]. The categories of absorption (poor, medium, excellent) indicate the relative ability of substances to be absorbed into the body. Poor absorption suggests challenges in absorption, while excellent absorption suggests efficient uptake. Based on this information, substances **15115**, **15068**, and **9335** demonstrate both excellent P-glycoprotein inhibition and absorption properties. This indicates that these substances have a high potential for absorption and distribution in the body. Substances **360799** and **22591** show medium P-glycoprotein inhibition and absorption, indicating a moderate ability to be absorbed. However, substances **10015** and **20988** exhibit poor P-glycoprotein inhibition and absorption characteristics, suggesting difficulties in their absorption and distribution within the body. Based on the provided data, all the compounds demonstrate excellent absorption, indicating a high likelihood of being effectively absorbed in the human intestine. However, the reference compound MEY exhibits medium absorption, suggesting a moderate potential for absorption. The bioavailability of a drug is an important pharmacokinetic parameter that assesses the fraction of an administered dose that reaches the systemic circulation in an unchanged form. It reflects the extent and rate of drug absorption into the bloodstream and influences its therapeutic effectiveness. The data suggest that all the compounds, including MEY, exhibit excellent permeability at a rate of 20%. After obtaining encouraging absorption results, the focus then shifted toward distribution analysis, aiming to gain valuable insights into the potential distribution patterns of these compounds across various tissues and compartments within the body.

#### 2.3.4. Distribution

Continuing with the analysis, the predicted distribution results provide valuable insights into the potential tissue and compartmental distribution of the Hsp90 inhibitors under investigation (Table 6). By understanding their distribution patterns, we can discern how these compounds may interact with specific target tissues or organs, thus informing their pharmacological activity and potential therapeutic effects. The provided values for PPB (percentage of protein binding) represent how much a compound binds to plasma proteins. A compound is considered to have significant protein binding if its predicted value is equal to or greater than 90%. After analyzing these values, it is clear that substances **10015**, **360799**, and MEY have high levels of protein binding, indicating that a substantial portion of these compounds bind to plasma proteins. In contrast, the remaining substances have values that are close to 90%. It is crucial to recognize that drugs with high protein binding may have a low therapeutic index since a smaller amount of the drug remains in its active, unbound form.

The predicted VD values for the compounds indicate their estimated volume of distribution in L/kg. A proper VD is typically within the range of 0.04–20 L/kg. The given compounds, including substances **10015**, **360799**, **15115**, **20988**, **15068**, **9335**, and **22591**, all have predicted VD values that fall within this acceptable range. This suggests that these compounds have an appropriate extent of distribution throughout the body. The BBB penetration values provided indicate the ability of molecules to cross the blood—brain barrier. Analyzing the given values, substances **360799**, **20988**, **10015**, **15115**, **9335**, and **15068** are classified as excellent, indicating a higher probability of BBB penetration. On the other hand, compound **20988** is classified as poor, suggesting a lower probability of BBB penetration.

#### 2.3.5. Metabolism and Excretion

The ability of a compound to inhibit specific enzymes is an important factor to consider in drug development and clinical use. Inhibition of these enzymes can impact the metabolism and clearance of drugs, influencing their efficacy, safety, and potential for drug—drug interactions. Therefore, identifying the inhibitory effects of compounds on specific enzymes provides valuable information for understanding their pharmacokinetic profiles and optimizing their therapeutic use. Based on the results (Table 7), it is evident that each compound exhibits varying inhibitory effects on different enzymes. This variability in inhibition suggests that the compounds may be susceptible to inhibiting at least one of the enzymes, which can be considered a favorable outcome. Table 7 provides information on the clearance (CL) and half-life (T1/2) values for different compounds. Clearance represents the rate at which a substance is eliminated from the body, while half-life represents the time it takes for the concentration of a substance in the body to reduce by half. Upon analyzing the data, several observations can be made regarding the clearance (CL) and half-life (T1/2) of the substances under investigation. The unit of predicted clearance (CL penetration) is expressed in ml/min/kg. Clearances are categorized as follows: >15 mL/min/kg signifies high clearance, 5–15 mL/min/kg suggests moderate clearance, and <5 mL/min/kg indicates low clearance. In our analysis, substances **10015**, **360799**, and **9335** demonstrate low clearance, implying a slower rate of elimination from the body. In contrast, substances **15115**, **22591**, and **20**,**988** exhibit moderate clearance. Furthermore, the range of half-life (T1/2) values, spanning from 0.316 to 0.892, typically suggests an intermediate half-life. It is crucial to note that these values may be categorized in the database output as either category 1 (representing a long half-life, >3 h) or category 0 (representing a short half-life, <3 h). This observation indicates that these compounds are rapidly cleared from the body. It is important to consider that substances with low clearance and short half-lives are typically eliminated relatively quickly.

#### 2.3.6. Prediction of Toxicity

For the toxicity study, we utilized two online platforms to ensure a comprehensive analysis, as explained in Section 4.6. Our analysis commenced with the findings from AdmetLab, outlined in Table 8. Notably, concerning hERG blockers, all compounds were classified as non-hERG blockers. This classification indicates a reduced risk of inducing potentially serious cardiac arrhythmias associated with these compounds. Moving on to rat oral acute toxicity, compounds **10015** and **360799** demonstrate acute oral toxicity in rats. In contrast, compounds **15115**, **20988**, **9335**, and MEY exhibit non-acute oral toxicity in rats. These findings provide insights into the potential risks associated with the ingestion of these compounds. In examining skin sensitization, compounds **10015**, **360799**, **15115**, **9335**, and MEY are classified as non-skin sensitizers, indicating that they are unlikely to cause skin sensitization. However, compound **20988** shows potential for skin sensitization, suggesting the need for further investigation and consideration of its potential risks. In terms of respiratory toxicity, it was observed that compound **10015** displays respiratory toxicity, whereas the remaining compounds do not exhibit such effects. This information holds significant importance in the evaluation of potential adverse impacts on the respiratory system.

Table S3 shows the findings encompassing diverse organ toxicity and endpoints for the marine compounds selected, evaluated via the Protox-II toxicity analysis platform. For these results, the table delineates the accuracy percentage of predictions and the average similarity percentage in comparison to the datasets utilized by the models. The evaluation encompassed predictions for various toxicities, including human hepatotoxicity (H-HT)/drug-induced liver injury (DILI), carcinogenicity, immunotoxicity, mutagenicity, and cytotoxicity among the candidate compounds. Safety assessments indicated that most of the lead compounds exhibited promising safety profiles regarding H-HT/DILI. However, the reference compound, MEY, demonstrated a potentially considerable hepatotoxicity risk, with a safety profile score of 0.50.

Regarding carcinogenicity, compounds **22591** and **360799** were identified as noncarcinogenic (inactive). Conversely, the remaining compounds showed a low probability of carcinogenicity, ranging between 0.51 and 0.62. This likelihood might arise from the presence of primary and secondary amine functional groups in their structures, known to react with sodium nitrite, forming N-nitroso compounds that contribute to potential carcinogenic risk. Immunotoxicity was noted in the marine compounds, with compound **10015** predicted to be immunotoxic (active) at a probability of 0.55. In contrast, the other compounds, including the reference compound MEY, exhibited non-immunotoxic (inactive) properties, with probabilities ranging between 0.54 and 0.67.

Following an analysis of pharmacokinetic properties, it was decided to discontinue the study of compound **10015** due to its multiple indications of potential toxicity. Our attention was then redirected toward an in-depth exploration of the dynamics and pharmacological potential of the remaining compounds.

### 2.4. Molecular Dynamics Analysis

Molecular dynamics simulations using Gromacs software were conducted to verify the stability of Hsp90 and its complexes. In a specific solvation system, Hesp90 with its ligands (**15115**, **360799**, **20988**, **22591**, and **9335**) was simulated for 100 ns on a GPU system by quantifying the root-mean-square deviation (RMSD), root-mean-square fluctuations (RMSF), radius of gyration (R_g_), and solvent available surface area (SASA) of the active site of Hsp90 and its complexes.

#### 2.4.1. RMSD of HSP90 and Its Complexes

**Figure 5:**
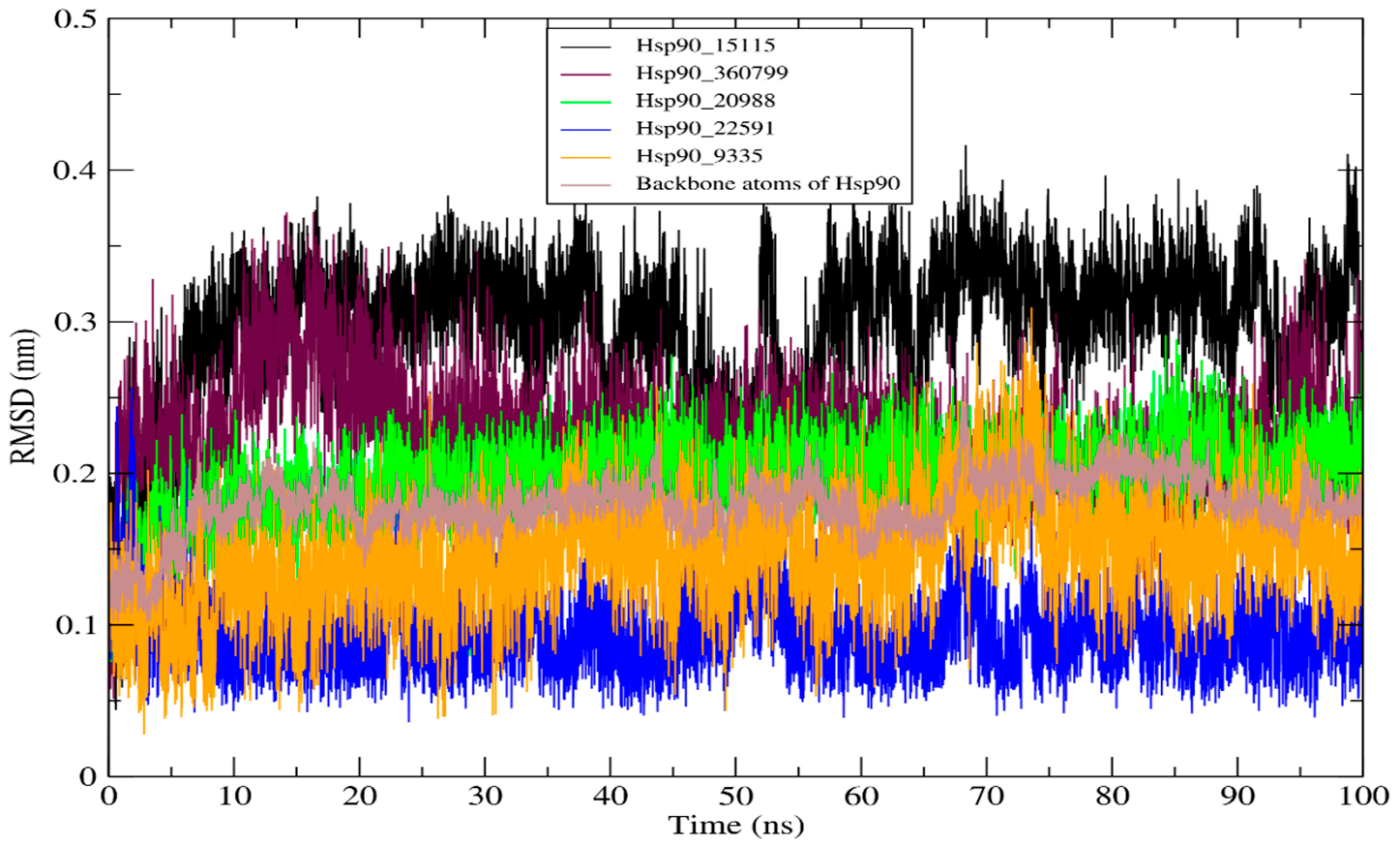
RMSD profile of Hsp90 and its complexes for 100 ns of the simulation period.

RMSD is a metric in MD simulations that measures the equilibrium stability and flexibility of proteins and ligands, as well as the distance that exists between the protein’s backbone and atoms [39]. The average RMSD values of backbone atoms for Hsp90 and its complexes with the hit molecules **15115**, **360799**, **20988**, **22591**, and **9335** were estimated to be 0.179, 0.295, 0.223, 0.195, 0.094, and 0.147 nm, respectively. Figure 5 demonstrates the average RMSD values of the backbone atoms Hsp90 and its complexes. The hit molecules **20988**, **22591**, and **9335** formed exceptionally stable complexes with the active site of Hsp90 over 100 ns of simulation, showing that the complexes generated have excellent structural and dynamical stability. The findings provide information on the stability of marine chemical compounds within the active site of Hsp90, which might be important in the development of novel powerful treatments for malignant tumors.

#### 2.4.2. RMSF of HSP90 and Its Complexes

**Figure 6:**
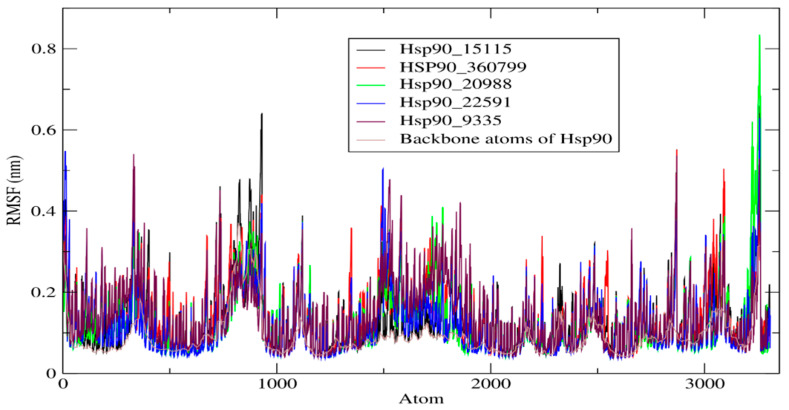
RMSF analysis graphical representation of backbone atoms of Hsp90 and its five complexes for 100 ns of simulation.

RMSF analysis was adopted to predict the shift in atom location for the backbone atoms of Hsp90 and their five complexes. A smaller RMSF value explained the stability of the Hsp90_hit molecule complexes, as opposed to a higher value reflecting more flexibility over the 100 ns simulation. Figure 6 depicts the RMSF fluctuation of molecular dynamic simulations for all systems. The analysis revealed that the average RMSF of the backbone atoms of Hsp90 was 0.093 nm. Furthermore, the average RMSF for all compounds ranged between 0.127 and 0.142 nm, suggesting a low level of atomic mobility. In particular, the backbone atoms of Hsp90 fluctuated more at atom 985, with an RMSF value of 0.6 nm. In addition, the Hsp90_9335, Hsp90_22591, and Hsp90_360799 complexes exhibited higher fluctuations at atoms 300, 1400, and 2800, with average RMSFs varying between 0.3 and 0.6 nm. We also noticed a similar dynamic movement toward the Hsp90 binding site, indicating a regular interaction between the hit molecules and Hsp90. In general, the RMSF study sheds light on the dynamics of Hsp90 and its complexes, which can be used to design new anticancer drugs.

#### 2.4.3. Radius of Gyration (Rg)

**Figure 7:**
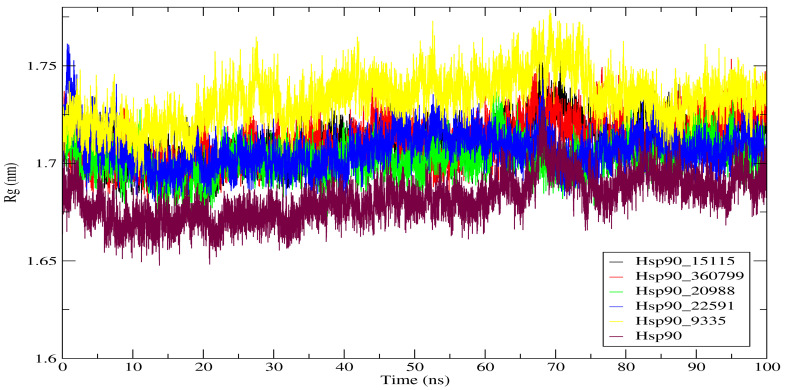
Radius of gyration (Rg) of Hsp90 and its complexes over 100 ns of simulation.

The radius of gyration (Rg) describes the variations in compactness of a protein—ligand complex. It refers to the unfolding and folding of proteins in molecular dynamic simulations. The average Rg for Hsp90 was 1.733 nm. All of the compounds had an average Rg between 1.707 and 1.733 nm. The Rg findings revealed that each Hsp90_hit molecule complex was stable with a similar compact to the reference protein (Hsp90). The similarity of the average Rg values of the five complexes to the average Rg of Hsp90 can be explained by the structural stability during the interaction period with the active site of Hsp90. This finding is in line with the RMSD and RMSF examinations that also showed that all the complexes were very stable over 100 ns. In Figure 7, we present the Rg values for each system plotted over the duration of the simulation.

#### 2.4.4. Solvent Accessible Surface Area (SASA) Analysis

**Figure 8:**
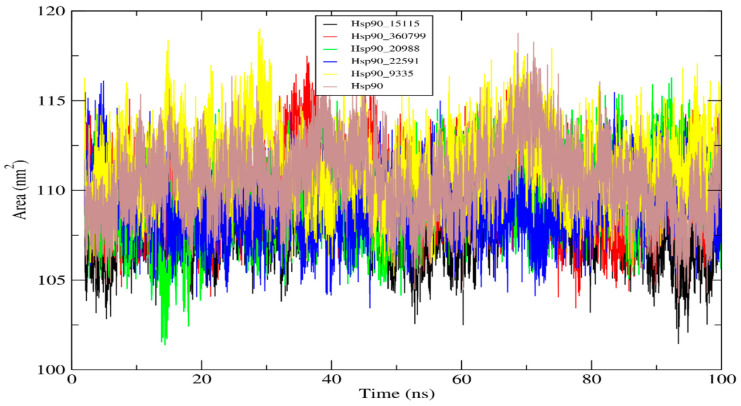
The graph representing the solvent accessible surface area (SASA).

SASA describes the interaction of Hsp90_hit compound complexes with solvents and corresponds to the solvent-accessible surface area. Furthermore, it predicts the structural changes that occur throughout the interactions. The Hsp90 protein exhibited an average SASA value of 110.61 nm^2^, whereas the average SASA values of its five complexes ranged from 109.26 to 111.44 nm^2^ (as indicated in Table 9). The SASA value of the five Hsp90_hit molecule complexes remained considerably stable over 100 ns of simulation, indicating no changes in the Hsp90 conformation. Furthermore, without altering its structure, Hsp90 is able to continue performing its essential functions without perturbations. Figure 8 depicts the changes in the solvent accessibility of the Hsp90_hit compound complexes during 100 ns of simulation.

#### 2.4.5. MM-PBSA Calculations

**Figure 9:**
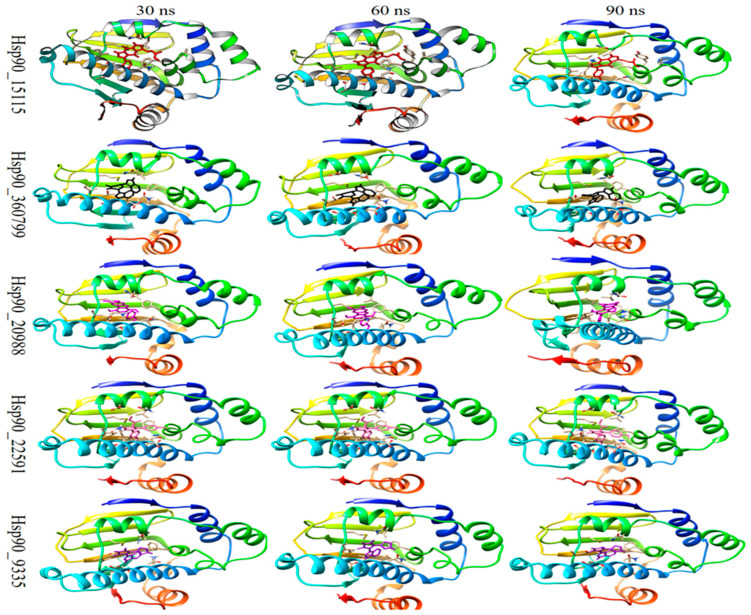
Binding poses of the five complexes over 100 ns of simulation.

The binding free energy at 100 ns was computed from the molecular dynamic trajectories via the MM-PBSA method developed in Gromacs. The calculated total binding free energy was computed based on the van der Waals interactions (ΔE_VDW_), electrostatic interactions (ΔE_EEL_), generalized born component (ΔE_GB_), nonpolar solvation component (ΔE_SURF_), total gas phase molecular mechanics energy (ΔG_GAS_), and total solvation energy (ΔG_SOLV_) for the five complexes. The total binding energies of the five complexes were found to be between −32.78 and −23.11 kJ/mol, as shown in Table 10. In addition, according to all MM-PBSA calculations, the five compounds formed stable complexes with the active site of Hsp90 in the same configuration as the docking investigation. The estimation of binding free energy corroborated the results of the molecular docking and molecular dynamic simulations. These findings may be useful in designing potent drugs for the treatment of incurable and malignant illnesses. Figure 9 represents the binding poses of each system over the simulation time.

## 3. Discussion

Hsp90 is a critical chaperone protein that interacts with cancer client proteins and co-chaperones to regulate signaling pathways and repair folded proteins in tumor cells [40]. As a result, it is an essential target in the treatment of cancer and its complications. The goal of our research was to identify novel and potent Hsp90 inhibitors utilizing natural marine compounds. The pharmacophore based on computer-aided technology was used in the initial phase to screen the CMNPD database for novel effective inhibitors. Docking modeling represents one of the computational strategies employed in drug development. It also predicts the affinity of compounds bound within protein receptors and generates an affinity score [41,42]. After examining the docking (XP) findings, the molecules CMNPD **22591**, **9335**, **15115**, **20988**, **10015**, and **360799** had XP scores ranging from −9.03 to −8.35 kcal/mol, whereas the reference molecule (MEY) had a score value of −8.32 kcal/mol. For the molecular interactions, specific amino acids, such as Gly97, Thr184, Asn51, and Asp93, were shown to be involved in hydrogen bonding interactions with the chosen compounds within the Hsp90 receptor during molecular interactions. These findings are consistent with the observations made in Abbasi et al. [28,33]. It is important to note that these identical amino acids were also found in the reference ligand in complex with Hsp90 [43]. These docking results support and explain the potential of the hit molecules to block the biological and biomolecular activity of Hsp90 by binding with its active site. Concerning the pharmacokinetics of the proposed molecules, intestinal absorption is easy and rapid, permitting significant quantities of these compounds to enter the bloodstream, and oral administration is perfectly acceptable. Most importantly, all of the proposed molecules, with the exception of molecule **20988**, have a high affinity for biological membranes; as a result, all of these compounds reach all biological tissues and organs (excellent distribution). At the level of hepatic metabolism, the proposed compounds (excluding **360799**) and the reference molecule inhibit the reactions of hepatic enzymes such as the cytochrome p450 family. In addition, due to the high solubility of the hit molecules with logS between −5.348 and −3.337, these molecules will be easily eliminated via the renal pathway (at the nephron level) or the enterohepatic cycle. Finally, all of the above findings suggest that the pharmacokinetic features of the examined marine compounds are optimal. Molecular dynamics simulations have been employed to determine the stability of drugs that bind to protein receptors. The suggested marine compounds showed average RMSD values ranging from 0.147 to 0.295 nm, with an average RMSF between 0.127 and 0.142 nm demonstrating minimal atomic movement and the continuance of structural stability throughout 100 ns of simulation. Furthermore, the data obtained from the interpretations of the molecular radius of gyration and solvent accessible surface area study support the RMSD and RMSF investigations. This outcome demonstrates the extent of the biomolecular structural stability of the generated complexes, ensuring the inhibition of Hsp90’s critical role in cancer cells. The MM-PBSA calculations agree with the molecular docking analysis, demonstrating that all hit molecules form stable complexes with the Hsp90 receptor, with binding free energies ranging from −32.78 to −23.11 kJ/mol. Our biomolecular structure and dynamics findings agree with the findings of Priya Antony and colleagues, who showed that marine-derived compounds form extremely stable complexes with different proteins [44]. Therefore, our findings should encourage cancer researchers to develop powerful and novel anticancer drugs based on natural marine molecules. On the other hand, Hsp90 inhibitors can potentially enhance the efficacy of antibiotics or serve as a target for developing new antimicrobial drugs, playing a crucial role in protein folding and stability in various organisms, including marine organisms. Inhibition of Hsp90 can disrupt essential cellular processes, making it an attractive target for antimicrobial strategies [45]. Studies on this issue are scant, and consequently, we propose the undertaking of additional and forthcoming investigations in this field.

## 4. Materials and Methods

### 4.1. Preparation of Library of Natural Compounds

Approximately 31,488 compounds of marine origin were obtained from the CMNPD database (https://www.ibscreen.com/natural.shtml accessed 14 may 2023) and imported into the Maestro molecular interface, which is part of the Schrödinger suite, for computational analysis. To prepare the ligands for further studies, LigPrep software (LigPrep, released in 2018) from Schrödinger was employed to consider the possible ionization states of the ligands. In this study, ionization states were generated at a physiological pH of 7.2 ± 0.2. Default options were utilized for all other parameters. The next step involved minimizing the ligand structures using the optimized potential liquid simulation (OPLS3) force field [46]. This minimization process was applied to optimize the ligand structures and improve their energetics, making them suitable for subsequent molecular modeling studies or virtual screening experiments.

The drug ability of these compounds was predicted using certain criteria. In this study, the Lipinski violation was set to zero, following the guidelines proposed by Lipinski et al. in 1997 [35]. Additionally, the compounds were assessed for their gastrointestinal (GI) absorption potential, with a high GI absorption considered. Based on these criteria, a subset of 11,118 compounds was screened for further analysis.

### 4.2. Optimizing Protein Crystal Structures

The crystal structure of the human Hsp90 protein (PDB code: 3OWD with a resolution of 1.63 Å) was obtained from the Protein Data Bank. *N*-{[1-(5-chloro-2,4-dihydroxyphenyl)-2-oxo-2,3-dihydro-1*H*-benzimidazol-5-yl]methyl}naphthalene-1-sulfonamide (MEY) was the co-crystallized ligand in the inhibitor site of Hsp90. The co-crystallized ligand, *N*-Aryl-benzimidazolones (MEY), linked to the protein structure, has been recognized for its capability to inhibit enzymatic Hsp90 activity [47]. This characterization positions it as a crucial reference in our ongoing investigation. To resolve specific errors, such as missing hydrogen atoms resulting from X-ray crystallography, the protein structure was refined using the protein preparation wizard in Glide software (Schrödinger suite 2018-1). The refinement process involved removing unwanted molecules, such as water and heteroatoms, to ensure a purified protein structure. Steric conflicts and incorrect bond lengths and angles were corrected to optimize the overall quality and geometry of the protein structure. In addition, the partial charges of protein atoms were determined using force-field-based calculations. This involved assigning appropriate charges to each atom based on force-field parameters, ensuring an accurate representation of the electrostatic properties of the protein. In the last refinement step, a root-mean-square deviation (RMSD) threshold of 0.3 for heavy atoms was defined, and a full energy optimization was performed using the OPLS3 force field. This optimization step further improved the stability and energetics of the protein structure.

### 4.3. Molecular Docking Studies

The receptor grid generating panel in Glide was used to create the Glide grid file. The grid’s location was chosen based on the co-crystallized ligand that is present in the protein’s active site. After the docking calculations, the poses or conformations of the ligands with the highest binding energies were visualized using BIOVIA Discovery Studio Visualizer (Dassault Systems, San Diego, CA, USA).

### 4.4. Pharmacophore Modeling and Enrichment Study

In the study, a pharmacophore model was generated using the “Develop Pharmacophore from protein—ligand complex” option within the Phase module, with default parameters being applied. To assess the effectiveness of the generated model, we conducted an enrichment study involving the combination of a decoy set obtained from the Schrödinger database with 35 known actives identified from the literature [48]. The chemical structures of the actives were drawn in ChemDraw Ultra 10.0, and their SMILES representations were copied to OpenBabelGUI (https://openbabel.org/wiki/OpenBabelGUI accessed 17 November 2023) to convert them into a single SDF file. Following the execution of the study, the results were analyzed by generating a receiver operating characteristic curve (ROC) based on the ratio of correctly identified actives to decoys as per the pharmacophore model. The area under the curve (ROC-AUC) was calculated to gauge the model’s detection capability. A valid pharmacophore model typically exhibits an AUC > 0.5, with excellent detection capacity approaching 1. A higher early enrichment factor at these percentages indicates a better ability to identify true positives early in the screening process.

### 4.5. Virtual Screening

The pharmacophore model was then utilized to conduct virtual screening of the Marine Natural Products (CMNP) database. The CMNP database initially contained a total of 31,488 molecules. After applying the Lipinski rules, which evaluate the drug-likeness of compounds, a subset of 11,118 molecules that met the criteria were included for further analysis. By employing the pharmacophore models, the study identified and selected 5156 compounds from the CMNP database that matched the pharmacophore features.

### 4.6. In Silico and ADME-Tox and Drug-Likeness Prediction

After identifying the best performing compounds from the docking results, our focus shifted to examining their molecular characteristics and evaluating the ADME (absorption, distribution, metabolism, and excretion) properties. We utilized the ADMETlab 2.0 web servers for this purpose. Toxicity studies were conducted using both Protox-II and ADMETlab 2.0 web servers. The ADMETlab 2.0 server (https://Pharmaceutical, accessed 9 July 2023, Fronts Vol. 4 No. 4/2022 © 2022) and the Protox-II web server (https://tox-new.charite.de) were accessed for this analysis.

### 4.7. Molecular Dynamics Investigation

Molecular dynamic simulations were utilized to analyze the protein—ligand complexes according to various physiological circumstances [49]. The molecules **15115**, **360799**, **20988**, **22591**, and **9335** were selected for MD simulations owing to their high binding with the Hsp90 active site. On the Ubuntu Linux distribution (v 24.04), Gromacs-2023 was employed to run molecular dynamics simulations over 100 ns for each system. The molecular topology file of the ligands was retrieved via the SwissParam online service, and the CHARMM27 all-atom force field was applied to generate the protein topology file [50,51]. The TIP3P water model was subsequently utilized to solve each system, and Na^+^ and Cl^−^ ions were introduced to neutralize the charge [52]. The solvated system was then used to minimize energy using the steepest descent minimization algorithm when the maximal force was below 10.0 kJ/mol. For the NVT equilibration period, the system was coupled using a v-rescale algorithm at 300 K with an initial coupling value of 0.1 ps and duration of 100 ps. The NPT was equilibrated for 100 ps utilizing a Berenson pressure-coupling procedure and a coupling constant of 2.0 ps [53,54]. The molecular dynamics simulation results were evaluated to assess the stability, mobility, compactness, and affinity of the five compounds with the active site of Hsp90. After the simulation, the structural stability of the Hsp90_hit molecule complexes was determined using root-mean-square deviation (RMSD). To assess the flexibility of the amino acid residues of each system, the root-mean-square fluctuation (RMSF) was computed. In addition, the radius of gyration (R_g_) was estimated to analyze the compactness of the Hsp90_hit complexes. Finally, the solvent accessible surface area (SASA) was calculated to check the stability of each system.

### 4.8. MM-PBSA Binding Free Energy Calculations

The molecular mechanics Poisson–Boltzmann surface area (MM-PBSA) and binding free energy investigations were carried out with the gmx_MMPBSA module of Gromacs-2023. The binding-free energy (ΔGbind) was determined in the following manner [55,56]:

ΔGbind = Gcomplex − (Gprotein + Gligand)

where ΔGbind is the overall binding energy of the complex, Gcomplex is the binding energy of Hsp90, and Gligand is the binding energy of the ligand.

## 5. Conclusions

Our research was dedicated to the pursuit of novel inhibitors for the Hsp90 protein, starting from a diverse collection of marine-derived compounds. Through our study, we successfully identified five marine-derived compounds, denoted as compounds **22591**, **9335**, **360799**, **15115**, and **20988**. These compounds are notable not only for their structural diversity, which encompasses stilbenes, indoles, pyrroloindoles, and phenylbenzofurans, but also for their marine origins, spanning from demosponges and *Didemnum granulatum* to tunicate *Rhopalaea* sp., *Spongosorites* sp., *Zyzzya fuliginosa*, and *Penicillium chermesinum*. Remarkably, despite their structural variances and marine sources, all of these compounds exhibited highly promising interactions with the Hsp90 protein. They formed multiple hydrogen bonds with the protein, thus significantly enhancing its stability. Moreover, we ensured the safety of these compounds through ADMETOX analysis. What strengthened the potential candidacy of our compounds as pharmaceutical agents was their stability within the challenging protein complexes, as confirmed through an in-depth study using molecular dynamics simulations. This research not only advances our understanding of Hsp90 inhibition but also paves the way for the development of potential therapeutic agents. The stability and interaction profiles of these marine-derived compounds demonstrate their promise in further drug-development efforts, particularly in the fields of anticancer and antimicrobial activity.

## Figures and Tables

**Table 1:** Docking binding energy (kcal/mol) of the top compounds according to the SP and XP methods.

Compound CMNPD	SP (kcal/mol)	XP (kcal/mol)
**22591**	−8.97	−9.03
**9335**	−9.04	−8.98
**15115**	−8.71	−8.73
**20988**	−8.86	−8.43
**10015**	−7.91	−8.35
**360799**	−7.62	−8.74
MEY	−8.84	−8.32

**Table 2:** Type of interaction and amino acid residues involved in that interaction inside the binding pocket of the Hsp90 enzyme (3owd).

Compound Name	H-Bonds			Hydrophobic		Others
	Amino Acid Residues	Number of H-Bonds	H-Bond Distance(Å)	Amino Acid Residues	Number of Hydrophobic Bonds	
** 10015 **	Gly97, Thr184, Asn51, Asp93	6	1.83–2.77	Ala55, met98	3 3	**/**
** 15115 **	Gly137, Phe138, Gly97, Leu48, Ser52, Asn106, Val136	10	2.10–2.92	Ala55	2	**Met98**
** 360799 **	Gly97, Thr184, Asp93	6	1.83–2.75	Ala55, Val86	2	**Met98**
** 20988 **	Gly97, Asn102, Leu48	3	1.83–2.51	Ala55, Val186	2	**Met98, Lys58**
** 22591 **	Gly97, Thr184, Leu48, Asn51	6	2.05–2.76	Ala55, Met98	2	**Met98**
** 9335 **	Glu97, Thr98, Asp39, Asn51	7	1.90–2.91	Ala55	2	**Met98**
**MEY**	Thr184, Gly97, Asp54	4	1.70–3.05	Phe138, Lys58, Met98, Leu108, Ala55	7	**/**

**Table 3:** The molecular properties of top marine compounds.

	10015	360799	15115	20988	9335	22591	MEY
**Molecular Weight (MW)**	354.1	328.1	363.12	338.0	278.08	369.12	490.07
**Volume**	358.9	338.1	362.03	331.96	271.60	368.87	459.9
**nRot**	3	2	5	2	2	6	5
**nRing**	5	5	4	4	4	3	5
**nHet**	6	5	7	6	6	7	10
**Flexibility**	0.017	0.074	0. 107	0.095	0.091	0.316	0.167
**TPSA**	90.11	73.04	106.26	103.2	90.64	105.09	124.42
**logS**	−4.645	−4.511	−5.348	−4.29	−3.630	−3.337	−4.776
**logP**	2.611	3.404	2.503	3.869	1.182	2.938	4.562
**logD**	2.616	3.172	2.100	3.060	1.413	2.931	3.492

**Table 4:** The medicinal chemistry properties of top marine compounds.

	10015	360799	15115	20988	9335	22591	MEY
**QED**	0.389	0.510	0.472	0.411	0.455	0.620	0.293
**SAscore**	2.821	3.253	3.206	2.592	2.715	2.635	2.615
**Pfizer Rule**	Accepted	Rejected	Accepted	Accepted	Accepted	Accepted	Accepted
**GSK Rule**	Accepted	Accepted	Accepted	Accepted	Accepted	Accepted	Rejected
**Golden Triangle**	Accepted	Accepted	Accepted	Accepted	Accepted	Accepted	Accepted
**PAINS**	0 alert	0 alert	1 alert	0 alert	0 alert	0 alert	0 alert
**BMS Rule**	0 alert	0 alert	0 alert	1alert	0 alert	0 alert	0 alert
**Chelator Rule**	0 alert	0 alert	0 alert	0 alert	0 alert	0 alert	0 alert

**Table 5:** In silico prediction of absorption of top marine compounds.

Absorption	10015	360799	15115	20988	9335	22591	MEY
**Caco-2 Permeability**	−5.172	−5.112	−5.323	−4.694	−5.200	−4.621	−5.856
**MDCK Permeability**	5.2 × 10^-6^	4.5×10^-6^	5.4×10^-6^	2.8×10^-5^	6.9 ×10^-6^	1.5 ×10^-5^	1.3 ×10^-5^
**Pgp-inhibitor**	Poor	medium	excellent	poor	excellent	excellent	medium
**HIA**	excellent	excellent	excellent	excellent	excellent	excellent	medium
**F20%**	excellent	excellent	excellent	excellent	excellent	excellent	excellent

**Table 6:** In silico prediction of distribution of top marine compounds.

Distribution	10015	360799	15115	20988	9335	22591	MEY
**PPB**	98.924%	95.931%	86.725%	88.424%	88.245%	86.444%	98.622
**VD L/kg**	0.345	0.966	1.747	0.459	1.131	0.460	0.429
**BBB Penetration**	excellent	excellent	excellent	poor	excellent	excellent	excellent
**Fu**	0.998%	3.499%	14.958%	2.918%	18.876%	8.108%	0.663

**Table 7:** In silico prediction of metabolism and excretion of marine compounds.

Metabolism	10015	360799	15115	20988	9335	22591	MEY
**CYP1A2 inhibitor**	yes	yes	yes	yes	yes	yes	yes
**CYP2C1 inhibitor**	yes	yes	yes	yes	yes	yes	yes
**CYP2C9 inhibitor**	yes	yes	yes	yes	no	yes	yes
**CYP2D6 inhibitor**	no	no	yes	no	yes	no	yes
**CYP3A4 inhibitor**	yes	yes	yes	yes	yes	yes	yes
**Excretion**							
**CL ml/min/kg**	1.592	2.302	10.457	13.908	3.311	10.986	4.720
**T1/2**	0.693	0.408	0.316	0.842	0.895	0.892	0.322

**Table 8:** In silico prediction of toxicity of top marine compounds.

	10015	360799	15115	20988	9335	22591	MEY
**hERG Blockers**	no	no	no	no	no	no	no
**Rat Oral Acute Toxicity**	yes	yes	no	no	no	no	no
**Skin Sensitization**	no	no	no	yes	no	no	no
**Respiratory Toxicity**	yes	no	no	no	no	no	no

**Table 9:** Analysis of the stability of the Hsp90 protein and its complexes via molecular dynamics simulations.

Complexes	RMSD (nm)	RMSF (nm)	Rg (nm)	SASA (nm^2^)
**Hsp90_15115**	0.295	0.136	1.711	109.26
**Hsp90_360799**	0.223	0.142	1.711	110.08
**Hsp90_20988**	0.195	0.130	1.702	109.44
**Hsp90_22591**	0.094	0.127	1.707	109.26
**Hsp90_9335**	0.147	0.141	1.733	111.44
**Hsp90**	0.179	0.093	1.733	110.61

**Table 10:** The binding energy components calculated by the MM-PBSA approach.

Ligand_Hsp90 Complexes	ΔEVDW (kJ/mol)	ΔEEEL (kJ/mol)	ΔEGB (kJ/mol)	ΔESURF (kJ/mol)	ΔGGAS (kJ/mol)	ΔGSOLV (kJ/mol)	ΔTOTAL (kJ/mol)
**Hsp90_15115**	−34.59	−33.26	48.63	−5.39	−67.85	43.23	−24.61
**Hsp90_360799**	−34.89	−21.88	36.59	−4.70	−56.77	31.89	−24.88
**Hsp90_20988**	−35.58	−26.15	43.70	−5.08	−61.73	38.62	−23.11
**Hsp90_22591**	−41.86	−29.51	44.06	−5.47	−71.37	38.59	−32.78
**Hsp90_9335**	−29.83	−45.69	52.75	−4.17	−75.51	48.57	−26.94

## Data Availability

Data are contained within the article.

## Supplementary Materials

The following supporting information can be downloaded at: https://www.mdpi.com/1420-3049/28/24/8074/s1.
