# Supplementary material for: Marine-Derived Compounds as Potential Inhibitors of Hsp90 for Anticancer and Antimicrobial Drug Development: A Comprehensive In Silico Study
Source: Molecules. 2023 Dec 13;28(24):8074. doi: 10.3390/molecules28248074 (PMC10871121; doi:10.3390/molecules28248074)
Supplement: Supplementary file 1 [file molecules-28-08074-s001.zip › molecules-2730234-supplementary.pdf]

Marine-Derived Compounds as Potential Inhibitors of Hsp90 for Anticancer and Antimicrobial Drug Development: A Comprehensive In Silico Study

Mebarka Ouassaf, Lotfi Bourougaa, Samiah Hamad Al-Mijalli, Emad M. Abdallah, Ajmal R. Bhat, Sarkar M. A. Kawsar

Table S1. Properties and Definitions of Selected Marine-Derived Compounds as Potent Inhibitors of Hsp90 Protein.

**CMNPD**	**22591**	**9335**	**10015**	**360799**	**15115**	**20988**
**Structure**	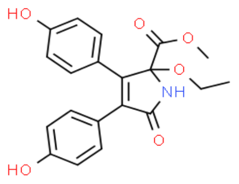	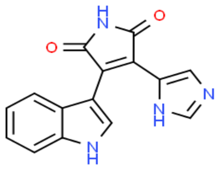	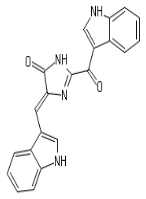	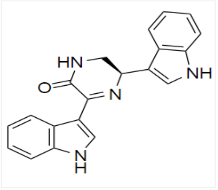	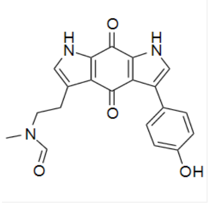	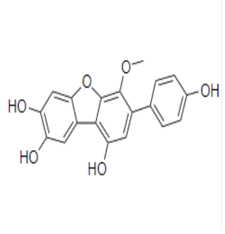
**Canonical SMILES**	c1cc(ccc1O)C2=C([C@](OC C)(NC2=O)C(OC)=O)c3ccc(O)cc3	C1(=O)NC(=O)C(c2c[nH]c(cccc3) c23)=C1c4[nH]cnc4	C1=CC=C2C(=C1)C(=CN2)C(=O)C3=N C(=C(N3)C=C4C=NC5=CC=CC=C54)O	c12c(cccc1)[nH]cc2[C@@H]3CNC (=O)C(c4c[nH]c(c4ccc5)c5)=N3	CN(CCC1=CNC2=C1C(=O)C3=C(C2=O)NC=C3C4=CC=C(C=C4)O)C=O	COC1=C2C(=C(C=C1C3=CC=C(C=C3)O)O)C4=CC(=C(C=C4O2)O)O
**PubChem CID**	162904707	398493	136691197	16104844	11717517	53262759
**Molecular formula**	C20H15NO6	C15H10N4O2	C21H14N4O2	C20H16N4O	C20H17N3O4	C19H14O6
**IUPAC name**	methyl (2R)-2-ethoxy-3,4-bis(4-hydroxyphenyl)-5-oxo-1H-pyrrole-2-carboxylate	3-(1H-imidazol-5-yl)-4-(1H-indol-3-yl)pyrrole-2,5-dione	[4-hydroxy-5-(indol-3-ylidenemethyl)-1H-imidazol-2-yl]-(1H-indol-3-yl)methanone	((3R)-3,5-bis(1H-indol-3-yl)-2,3-dihydro-1H-pyrazin-6-one	N-[2-[3-(4-hydroxyphenyl)-4,8-dioxo-1,7-dihydropyrrolo[3,2-f]indol-5-yl]ethyl]-N-methylformamide	3-(4-hydroxyphenyl)-4-methoxydibenzofuran-1,7,8-triol
**Synonym**	Ianthellidone B	Didemnimide A	Rhopaladins D	(R)-6′-debromohamacanthin B	Zyzzyanone D	3″-deoxy-6′-O-desmethylcandidusin B
**Resource**	Demospongiae	Didemnum granulatum	tunicate Rhopalaea sp	Spongosorites	Zyzzya fuliginosa	Penicillium chermesinum

Classy fire subclass	Stilbenes	Indoles	Indoles	Indoles	Pyrroloindoles	Phenylbenzofurans

Table S2. Result Of Enrichment Study.

Pharmacophore model	AUC	RIE	ROC	BedROC160.9	Total active	Number of ranked actives
DDRRR	0.84	7.18	0.78	0.58	35	29

Table S3. Organ and End Points Toxicity for Top Leads Compound.

			22591		9335		10015		360799		15115		20988		MEY	
			Probability	Prediction	Probability	Prediction	Probability	Prediction	Probability	Prediction	Probability	Prediction	Probability	Prediction	Probability	Prediction
Organ toxicity	Hepatotoxicity	dili	0.50	inactive	0.51	inactive	0.50	inactive	0.72	inactive	0.62	inactive	0.67	inactive	0.55	active
Toxicity end points	Carcinogenicity	carcino	0.58	inactive	0.51	active	0.55	active	0.56	inactive	0.59	active	0.62	active	0.71	inactive
	Immunotoxicity	immuno	0.95	inactive	0.73	inactive	0.7	inactive	0.98	inactive	0.79	inactive	0.8	inactive	0.54	inactive
	Mutagenicity	mutagen	0.67	inactive	0.61	inactive	0.6	active	0.67	inactive	0.54	inactive	0.55	inactive	0.78	inactive
	Cytotoxicity	cyto	0.66	inactive	0.7	inactive	0.74	inactive	0.77	inactive	0.55	inactive	0.95	inactive	0.64	inactive

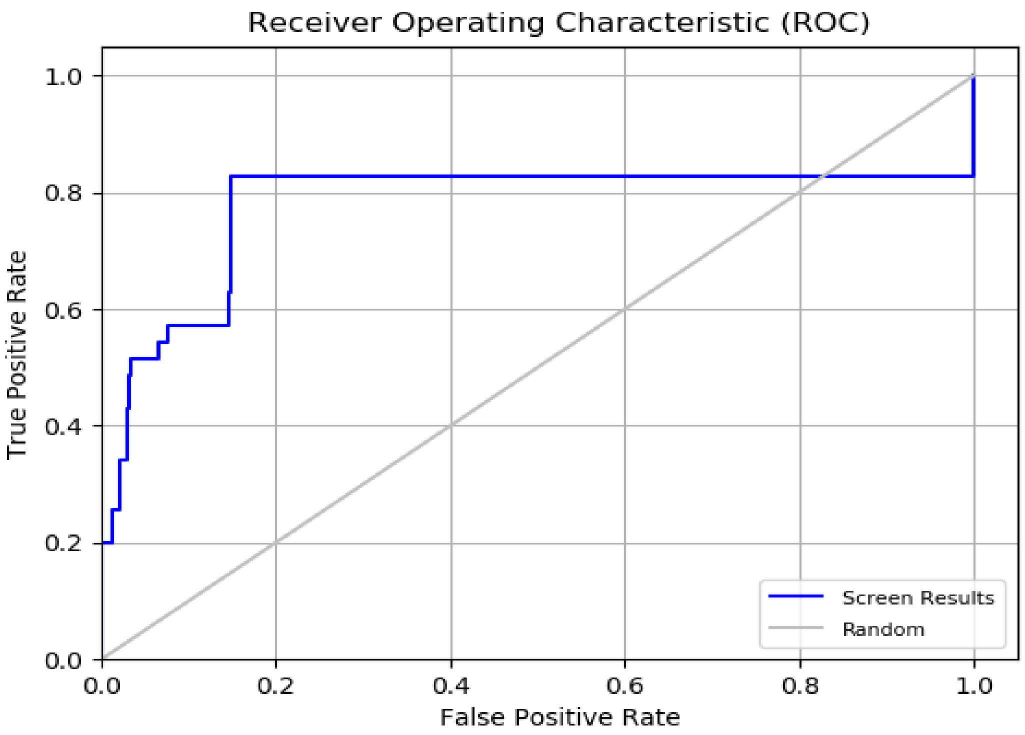

Figure S1. ROC plot of the pharmacophore model.

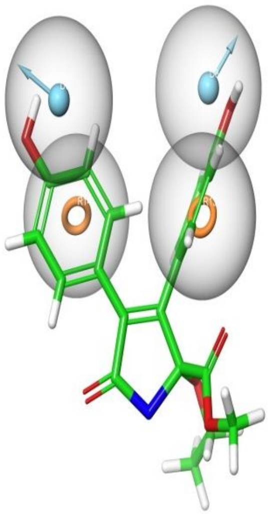

22591

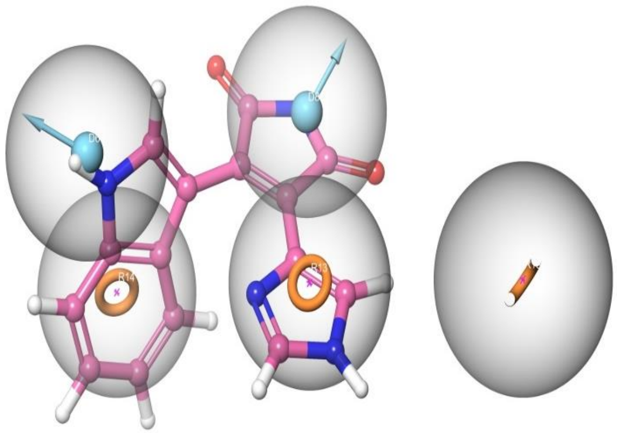

9335

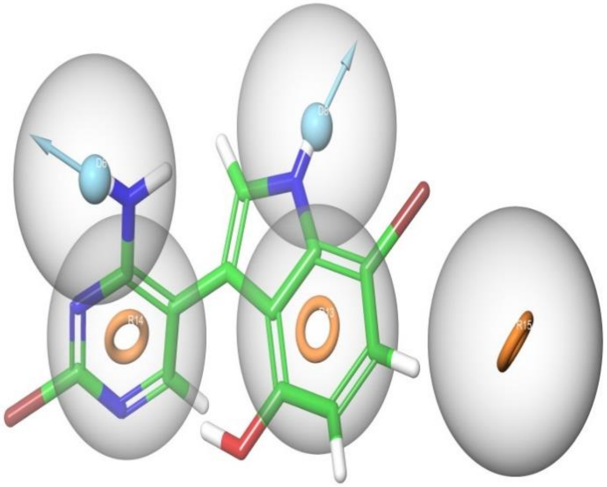

15115

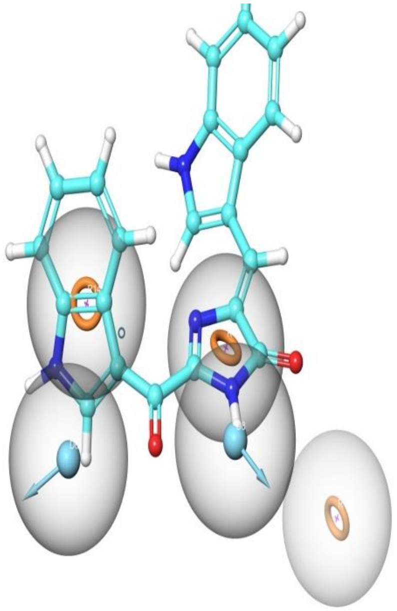

10015

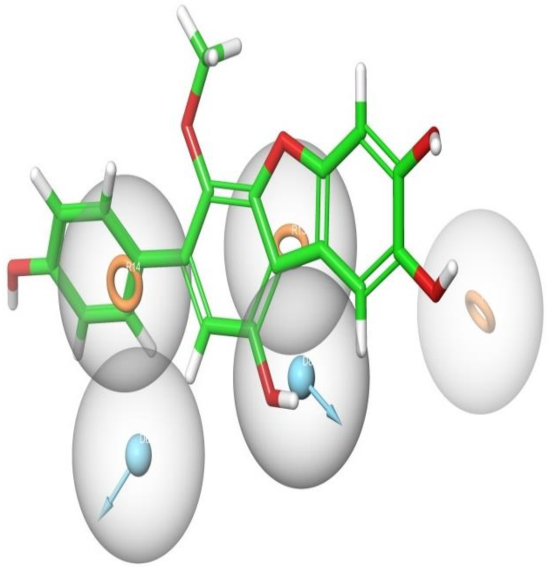

20988

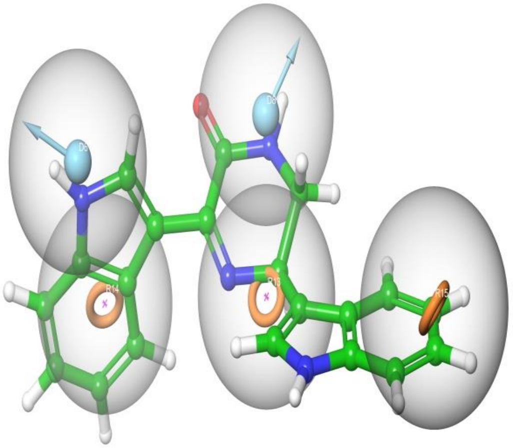

360799

Figure S2. The model map onto top leads.
